# Correction: Khan et al. The Extraction and Impact of Essential Oils on Bioactive Films and Food Preservation, with Emphasis on Antioxidant and Antibacterial Activities—A Review. *Foods* 2023, *12*, 4169

**DOI:** 10.3390/foods13182963

**Published:** 2024-09-19

**Authors:** Sohail Khan, Abdullah A. A. Abdo, Ying Shu, Zhisheng Zhang, Tieqiang Liang

**Affiliations:** 1 College of Food Science and Technology, Hebei Agricultural University, Lekai South Avenue, Baoding 071000, China; sohailkhan6566@yahoo.com (S.K.); drabdoabdullah2021@gmail.com (A.A.A.A.); hebaushuying@hebau.edu.cn (Y.S.); 2 Department of Food Science and Technology, Faculty of Agriculture and Food Science, Ibb University, Ibb 70270, Yemen; 3 Hebei Layer Industry Technology Research Institute, Economic Development Zone, Handan 545000, China

There was an error in the original publication [1]. A correction has been made to ref [30] and ref [91]; the updated references are as follows:

30. Gerbaud, V.; Rodriguez-Donis, I.; Hegely, L.; Lang, P.; Denes, F.; You, X. Review of extractive distillation. Process design, operation, optimization and control. *Chem. Eng. Res. Des.* **2019**, *141*, 229–271.
91. Mohamed, S.A.; El-Sakhawy, M.; El-Sakhawy, M.A.M. Polysaccharides, protein and lipid-based natural edible films in food packaging: A review. *Carbohydr. Polym.* **2020**, *238*, 116178.

The authors state that the scientific conclusions are unaffected. This correction was approved by the Academic Editor. The original publication has also been updated.
